# Optimisation of sodium and potassium concentrations and pH in the artificial seminal plasma of common carp *Cyprinus carpio* L.

**DOI:** 10.1007/s10695-018-0491-3

**Published:** 2018-03-21

**Authors:** Beata Irena Cejko, Ákos Horváth, Timea Kollár, Eszter Kása, Jelena Lujić, Zoran Marinović, Béla Urbányi, Radosław Kajetan Kowalski

**Affiliations:** 1 Department of Gamete and Embryo Biology, Institute of Animal Reproduction and Food Research, Polish Academy of Science, Olsztyn, Poland; 2 Department of Aquaculture, Institute of Aquaculture and Environmental Safety, Szent István University, Gödöllő, Hungary

**Keywords:** Common carp, Sodium, Potassium, pH, Artificial seminal plasma, Sperm preservation, Chilled storage

## Abstract

The effect of sodium and potassium concentrations as well as optimal pH on the motility of common carp *Cyprinus carpio* L. sperm during short-term storage in artificial seminal plasma (ASP) was investigated. Sperm was collected from individual males (*n* = 5) and each sample diluted tenfold (1:9) in ASP (sperm:extender) containing 2 mM CaCl_2_, 1 mM Mg_2_SO_4_ and 20 mM Tris at pH 8.0 and supplemented by the following concentrations of sodium and potassium (mM/mM): 0/150, 20/130, 40/110, 75/75, 110/40, 130/20 and 150/0. The osmolality of all ASP variants was set at 310 mOsm kg^−1^. Sperm motility was measured using a CASA system during 72 h of storage. Immediately after dilution, sperm motility was high (90%) both in each variant and in the control group (fresh sperm). After 72-h storage, the highest sperm motility was noted in ASP containing 110 mM NaCl and 40 mM KCl. No differences were found in the motility of samples preserved within the pH range of 7.0–9.0. Our data suggest that for the short-term storage of common carp sperm, whereas the pH of the solution does not play a crucial role, a specific potassium concentration of around 40 mM is required.

## Introduction

The most important sperm parameter affecting fertilisation capacity in fish is sperm motility. The percentage of motile sperm (MOT, %) and curvilinear velocity of sperm (VCL, μm s^−1^) are considered the most important CASA parameters, since both directly affect the ability of sperm to fertilise eggs (Lahnsteiner et al. 1996; Gage et al. 2004). Moreover, in some fish species such as the European smelt *Osmerus eperlanus* (L.) and crucian carp *Carassius carassius* (L.), lateral head displacement (ALH, μm) may be associated with sperm maturity (Kowalski et al. 2006; Cejko et al. 2013a). Under natural conditions, seminal plasma (SP) maintains the ability of sperm to move and protects them during storage (in the sperm duct) against damage caused by reactive oxygen species (Rurangwa et al. 2004). It has also been postulated that the pH of SP has a significant influence on motility potential in some fish species (Lahnsteiner et al. 1996). Cations such as K^+^ and Na^+^ play a physical role in maintaining osmotic balance and are important components of a number of enzymes. Moreover, as K^+^ is a natural motility inhibitor in salmonid species, its high concentration in SP decreases sperm metabolism (Alavi and Cosson 2006). It has been confirmed that in salmonids and sturgeons, ions such as K^+^, rather than osmolality, are an essential factor in sperm motility inhibition (Morisawa and Suzuki 1980). In contrast, cyprinid sperm motility is inhibited by the high osmolality of the seminal plasma, with potassium ions not essential to this process (Perchec-Poupard et al. 1997; Cosson 2004). However, concentrations higher than 150 mM of KCl or NaCl have been found to inhibit common carp sperm movement (Perchec et al. 1995; Billard et al. 1995). Despite the fact that potassium ions are not essential for the immobilisation of common carp sperm, their concentration in seminal plasma is very high. The ionic concentration and osmolality of seminal plasma have been recorded at 75 mM, 82.4 mM, 2.0 mM, 0.8 mM, 112 mM, and 302 mOsm kg^−1^ for Na^+^, K^+^, Ca^2+^, Mg^2+^, Cl^−^ and osmolality, respectively (Morisawa et al. 1983). Márián et al. (1997) more recently reported that the pH of common carp seminal plasma is around 8.28.

Fresh sperm (non-diluted) stored in vitro lose its motility and viability very rapidly. Under such conditions, the protective role of SP is not sufficient and thus negative effects on sperm quality during preservation may be observed. It is also less effective to utilise such sperm for fertilisation, since its motility typically does not exceed 50% 24-h post-storage (Bozkurt and Secer 2005). To maintain sperm viability and fertilisation capacity in vitro*,* an appropriate extender must be used, i.e. artificial seminal plasma (ASP) including the proper balance of ions, osmolality and pH. This technique can be applied in hatchery practice, for example, in the absence of synchronisation of ovulation and spermiation, or to reduce broodstock manipulation and workload during spawning. Moreover, during short-term storage, sperm may be analysed in detail and that exhibiting the highest motility may be chosen for future fertilisation. Considering all the above advantages, the short-term storage of sperm is of increasing importance for artificial reproduction in fish (Kowalski et al. 2014; Křišťan et al. 2014; Sarosiek et al. 2014).

In common carp, several buffers have been tested for the short-term storage of sperm, with variability in sperm quality observed. For example, in fish-ringer extender containing 130 mM NaCl, 13.5 mM KCl, 13.5 mM CaCl_2_ and 30 mM Tris at pH 8.5 and 235 mOsm kg^−1^ (Rana and McAndrew 1989), the motility of common carp sperm stored at + 5 °C dropped significantly from 81 to 29% over 24 h. In contrast, sperm stored under the same conditions in BWW buffer containing 95 mM NaCl, 48 mM KCl, 1.7 mM CaCl_2_ and 25 mM NaHCO_3_ at pH 8.8 and 326 mOsm kg^−1^ retained 88% motility (Yanagimachi 1978), with the inhibition of sperm motility due to surrounding high osmolality (approximately 300 mOsm kg^−1^). Perchec et al. (1995) also reported that common carp sperm placed in 30 mM Tris-HCl buffer containing 200 mM KCl at pH 8.0 may be successfully preserved until activation. Therefore, although potassium ions do not play a crucial role in common carp sperm motility inhibition, their presence is essential for sperm motility maintenance during the quiescent stage, as suggested by Morisawa et al. (1983). Although NaCl has similar properties to KCl, the recovery of motility is more rapid with the latter (Redondo-Müller et al. 1991).

An assessment of sperm motility taking into account the most important CASA parameters enables the tracking of specific changes in sperm movement during preservation. Furthermore, detailed characteristics of sperm tracking measured at specific intervals (hours or days) and under specific conditions (extender, pH or temperature) may be useful in developing an appropriate technique for sperm preservation. The aim of the present study was thus to investigate the effect of sodium and potassium concentrations as well as pH value on the motility of common carp sperm during short-term storage (+ 4 °C) in ASP.

## Materials and methods

### Origin and hormonal treatment of males

A broodstock of common carp (age 2+, weight 288 ± 86 g, standard length 220 ± 2 mm, *n* = 114) was grown and maintained in a recirculating aquaculture system at 24 °C with a 10-h dark–14-h light photoperiod at the Department of Aquaculture of Szent István University in Gödöllő, Hungary. Sperm was collected from individual males (*n* = 5). Spermiation was induced with an intraperitoneal injection of 4 mg/kg body weight of carp pituitary extract 24 h prior to the planned sperm collection.

### Sperm collection and dilution ratio test

Sperm were obtained via gentle abdominal massage and collected by micropipette, with special care taken to avoid any contamination (urine and faeces). After collection, each sperm sample was diluted in a different dilution ratio (sperm:extender), either × 2 (1:1), × 5 (1:4), × 10 (1:9), × 20 (1:19) or × 40 (1:39), in ASP containing 75 mM NaCl, 70 mM KCl, 2 mM CaCl_2_, 1 mM Mg_2_SO_4_ and 20 mM Tris at pH 8.0 (Lahnsteiner et al. 1996). Undiluted sperm was used as a control. After 72-h storage in a thin layer (Eppendorf tube) under anaerobic conditions (4 °C), the most promising sperm dilution variant was selected for further analysis.

### Sperm preservation

After identifying the best dilution ratio, i.e. × 10 (1:9), sperm was collected from a further five males and each sperm sample diluted in ASP containing various proportions of sodium and potassium. The base artificial seminal plasma contained 2 mM CaCl_2_, 1 mM Mg_2_SO_4_ and 20 mM Tris at pH 8.0 and was supplemented by one of the following concentrations of NaCl and KCl: 0 mM NaCl/150 mM KCl, 20 mM NaCl/130 mM KCl, 40 mM NaCl/110 mM KCl, 75 mM NaCl/75 mM KCl, 110 mM NaCl/40 mM KCl, 130 mM NaCl/20 mM KCl and 150 mM NaCl/0 mM KCl. The osmolality of all ASP was set at 310 mOsm kg^−1^. Sperm was diluted in ASP (sperm:extender) and stored in an Eppendorf tube at 4 °C for 72 h. After 72 h, the most promising variant of ASP was selected, with this variant then subjected to testing at different pH values (7.0, 7.5, 8.0, 8.5 and 9.0).

### CASA analysis

Sperm motility was measured using a computer-assisted sperm analysis (CASA) system (SpermVisionTM v. 3.7.4., Minitube of America, Venture Court Verona, USA) every 24 h during 72 h of sperm storage in ASP. For the analysis of sperm motility, 1 μl of sperm was mixed with 100 μl of activation solution, which included 10 mM Tris buffer containing 100 mM NaCl at pH 9.0 and osmolality 200 mOsm kg^−1^ (Cejko et al. 2013b). In order to prevent sperm from adhering to the glass surface, 0.5% bovine serum albumin (BSA) was also added. The CASA system was used to determine the following parameters: percentage of motile sperm (MOT, %), progressively motile sperm (PRG, %), curvilinear velocity (VCL, μm s^−1^), straight-linear velocity (VSL, μm s^−1^), amplitude of lateral head displacement (ALH, μm) and beat cross-frequency (BCF, Hz). Sperm motility was measured twice (duplicate measurements) for each sperm sample preserved in ASP, with the average of these two measurements taken for every ASP variant and for each of the analysed CASA parameters. During CASA analysis, the semen samples were kept on ice (± 4 °C).

### Statistical analysis

Mean and standard deviation (± SD) were determined for each of the CASA parameters analysed and for all samples stored in the different ASP variants. Differences between parameters were established via two-way repeated measures ANOVA (*α* = 0.05). Analyses were performed using GraphPad Prism 6.0 (GraphPad Software Inc., San Diego, CA, USA).

## Results

### Effect of dilution ratio on sperm storage in ASP

**Fig. 1:**
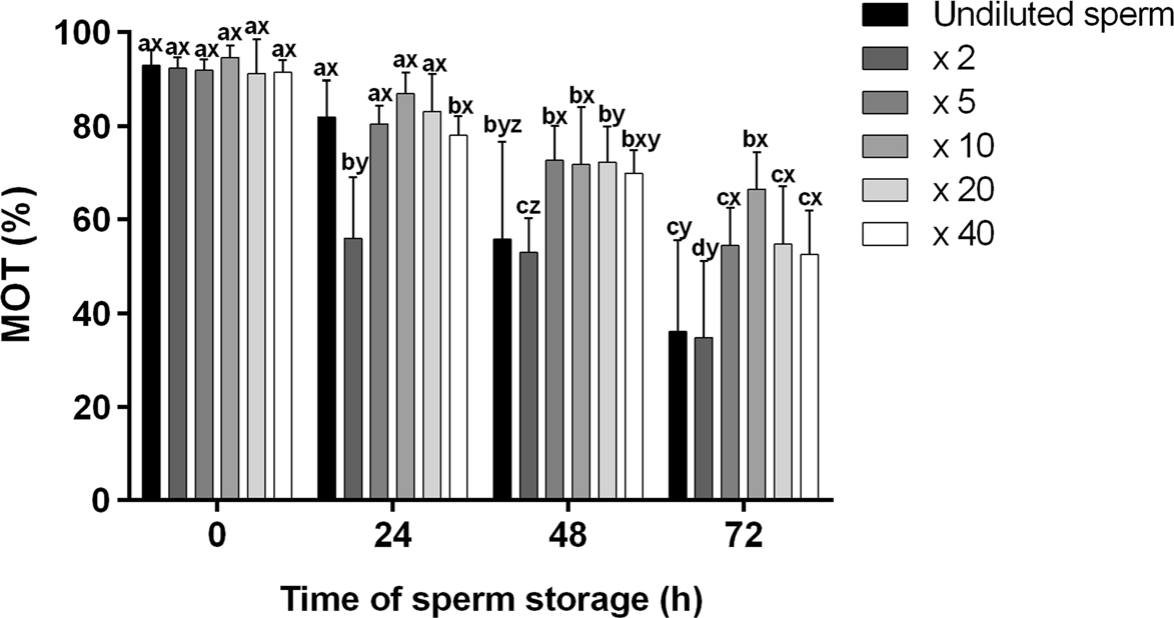
Effect of different dilution ratio (mean ± SD), i.e. × 2 (1:1), × 5 (1:4), × 10 (1:9), × 20 (1:19) and × 40 (1:39), on sperm motility (MOT, %) of common carp *Cyprinus carpio* L. during 72 h of short-term storage in ASP (75 mM NaCl, 70 mM KCl, 2 mM CaCl_2_, 1 mM Mg_2_SO_4_, 20 mM Tris at pH 8.0). Undiluted sperm was used as a control. Different superscripts, i.e. a, b, and c, indicate statistical differences between time points for the same dilution ratio whereas different superscripts, i.e. x, y, and z, indicate statistical differences between the dilution ratio at the same time point (*P* < 0.05)

Immediately after dilution (0 h), no differences in sperm motility (MOT, %) were observed among all variants (Fig. 1). After 24-h storage, sperm motility dropped to 60% in samples diluted × 2; similar results were observed in the control after 48-h storage. At this time, MOT values were at a constant level of around 80% in all other sperm dilution variants (× 5, × 10, × 20 and × 40). After 72-h storage, the highest motility was observed in sperm samples stored in ASP diluted at × 10 (75%), although this value differed significantly only from those recorded in the sample diluted × 2 and in the control (Fig. 1).

### Effect of ASP sodium and potassium concentrations

Immediately after dilution (0 h), sperm motility (MOT) was high (range 81.5–93.6%) in all ASP variants regardless of sodium and potassium concentrations, as well as in the control group (fresh sperm 98.4%) (Table 1). With the passage of time, sperm motility significantly dropped; after 72-h preservation, the lowest (12.8%) sperm motility was recorded in the ASP supplemented with 150 mM KCl without sodium addition. In contrast, the highest motility of sperm at this time was observed in the ASP containing 110 mM NaCl and 40 mM KCl (79.0%). A similar tendency was observed in the progressive motility of sperm, although in samples containing the highest concentrations of KCl (150, 130 and 110 mM) and the lowest concentrations of NaCl (0, 20 and 40 mM), PRG values did not exceed 10% (Table 1). After 72-h preservation, the greatest sperm velocity (VCL and VSL) was noted in ASP supplemented with 110/40 mM NaCl/KCl (113.5 and 95.1 μm s^−1^, respectively), although similar values (i.e. not statistically different) were also recorded in ASP containing 75/75, 130/20 and 150/0 mM NaCl/KCl (Table 1). In these ASP variants, VCL and VSL values did not differ significantly after 24-h preservation from those determined immediately after dilution (0 h) (Table 1). The highest values of ALH were found in fresh sperm (control) at time zero (2.63 μm); these values were also significantly different from those recorded for any combination of Na and K in ASP (Table 1; *P* < 0.05). After 72 h, no significant difference in ALH values was detected among the treatment groups and the control (Table 1). No variation in BCF was observed immediately after sperm dilution (0 h) in the various ASP variants (and in fresh sperm) and after 24-h sperm preservation. However, after 72 h, the greatest value of this parameter was found in ASP supplemented with 110/40 mM NaCl/KCl (27.1 Hz), with significantly lower values recorded in ASP supplemented with 0/150, 20/130 and 40/110 mM NaCl/KCl (Table 1; *P* < 0.05). The largest differences between lowest and highest CASA parameter values at 72-h preservation were noted for PRG (about 45.5 times), VSL (9.1 times), VCL (7.2 times), MOT (6.2 times) and BCF (5.7 times).

**Table 1:** Motility parameters of common carp *Cyprinus carpio* L. (*n* = 5) sperm during 72-h short-term storage in artificial seminal plasma (ASP: 2 mM CaCl_2_, 1 mM Mg_2_SO_4_, 20 mM Tris at pH 8.0) containing different concentrations of NaCl and KCl, and for the control group (fresh sperm)

CASA parameters	Time (hours)	Control	Sodium and potassium concentration in ASP						
			0 mM NaCl/150 mM KCl	20 mM NaCl/130 mM KCl	40 mM NaCl/110 mM KCl	75 mM NaCl/75 mM KCl	110mMNaCl/40 mM KCl	130 mM NaCl/20 mM KCl	150 mM NaCl/0 mM KCl
MOT (%)	0	98.4 ± 0.5^aA^	93.6 ± 5.1^aA^	91.1 ± 3.0^aA^	93.3 ± 4.0^aA^	92.5 ± 5.0^aA^	93.9 ± 4.2^aA^	91.1 ± 4.9^aA^	81.5 ± 9.4^aA^
	24	90.4 ± 7.6^aA^	62.5 ± 6.4^bB^	73.7 ± 12.4^abB^	72.3 ± 17.8^abB^	82.6 ± 6.1^aAB^	81.4 ± 7.4^aA^	89.4 ± 6.3^aA^	73.6 ± 14.0^abAB^
	48	37.5 ± 20.6^deB^	27.0 ± 8.6^eC^	45.9 ± 11.5^cdC^	47.1 ± 15.7^cdC^	73.5 ± 10.9^abBC^	88.3 ± 4.1^aA^	83.3 ± 6.8^abAB^	65.4 ± 12.9^bcBC^
	72	29.0 ± 23.6^cdB^	12.8 ± 8.9^dC^	21.2 ± 15.3^cdD^	31.9 ± 20.9^cC^	64.0 ± 9.3^abC^	79.0 ± 7.3^aA^	72.6 ± 9.8^abB^	54.2 ± 12.8^bC^
PRG (%)	0	93.9 ± 0.8^aA^	88.8 ± 6.5^aA^	85.2 ± 4.6^abA^	84.3 ± 6.2^abA^	82.6 ± 7.2^abA^	84.2 ± 3.9^abA^	69.3 ± 12.9^bcA^	58.4 ± 11.7^cA^
	24	69.8 ± 20.2^abB^	44.3 ± 11.9^cdB^	56.6 ± 17.6^bcB^	57.3 ± 14.5^bcB^	66.3 ± 8.7^abAB^	72.3 ± 9.6^abAB^	74.7 ± 12.4^aA^	39.0 ± 16.0^dB^
	48	17.2 ± 15.8^cC^	3.6 ± 4.7^cC^	9.8 ± 9.6^cC^	18.7 ± 14.0^bcC^	52.3 ± 5.4^aB^	67.0 ± 5.0^aB^	65.1 ± 12.1^aA^	29.6 ± 15.6^bB^
	72	8.0 ± 7.4^eC^	1.3 ± 1.9^eC^	2.1 ± 1.3^eC^	8.2 ± 7.6^deC^	38.2 ± 6.7^bcB^	59.1 ± 13.9^aB^	49.3 ± 10.6^bB^	24.3 ± 9.7^dC^
VCL (μm s^−1^)	0	131.7 ± 4.5^abA^	141.5 ± 2.2^abA^	149.6 ± 6.5^abA^	148.3 ± 6.6^abA^	151.4 ± 6.6^aA^	143.6 ± 5.9^abA^	135.9 ± 8.4^abA^	128.2 ± 6.9^bA^
	24	97.4 ± 24.6^cB^	112.4 ± 11.6^bcB^	121.2 ± 4.3^abB^	124.9 ± 7.8^abB^	133.6 ± 7.7^abA^	138.4 ± 4.5^aA^	135.5 ± 7.0^aA^	128.8 ± 11.4^abA^
	48	59.6 ± 26.1^bcC^	45.7 ± 13.8^cC^	62.1 ± 16.8^bcC^	76.8 ± 18.9^bC^	100.8 ± 18.9^aB^	107.1 ± 7.5^aB^	107.4 ± 6.1^aB^	104.7 ± 7.9^aB^
	72	51.7 ± 24.4^bC^	15.8 ± 14.9^cD^	50.8 ± 28.5^bC^	60.1 ± 14.7^bC^	100.6 ± 7.1^aB^	113.5 ± 9.9^aB^	103.1 ± 18.8^aB^	108.7 ± 17.6^aB^
VSL (μm s^−1^)	0	86.3 ± 18.0^cA^	118.6 ± 3.4^abA^	133.2 ± 5.3^aA^	130.5 ± 6.5^abA^	129.4 ± 12.9^abA^	121.7 ± 5.9^abA^	107.8 ± 8.6^bcA^	113.6 ± 9.2^abA^
	24	72.6 ± 15.7^bA^	102.1 ± 10.8^aA^	109.0 ± 5.0^aB^	111.6 ± 11.9^aA^	121.3 ± 8.3^aA^	119.6 ± 7.6^aA^	115.6 ± 13.0^aA^	115.5 ± 15.7^aA^
	48	46.7 ± 25.1^cB^	44.5 ± 9.7^cB^	51.4 ± 17.9^cC^	66.8 ± 18.6^cB^	85.6 ± 17.5^abB^	86.0 ± 13.4^abB^	88.4 ± 12.4^abB^	100.9 ± 10.2^aA^
	72	39.0 ± 23.9^bB^	11.1 ± 10.1^cC^	43.1 ± 31.2^bC^	48.6 ± 18.2^bB^	88.1 ± 8.4^aB^	95.1 ± 13.1^aB^	84.3 ± 17.7^aB^	101.1 ± 15.9^aA^
ALH (μm)	0	2.63 ± 0.7^aA^	1.76 ± 0.3^bA^	1.55 ± 0.4^bA^	1.56 ± 0.2^bA^	1.71 ± 0.4^bA^	1.72 ± 0.3^bA^	1.76 ± 0.3^bA^	1.46 ± 0.2^bA^
	24	1.79 ± 0.5^aB^	1.51 ± 1.4^aA^	1.49 ± 0.6^aA^	1.45 ± 0.2^aA^	1.47 ± 0.3^aA^	1.56 ± 0.3^aA^	1.65 ± 0.3^aA^	1.51 ± 0.2^aA^
	48	1.29 ± 0.3^aB^	1.26 ± 0.4^aA^	1.42 ± 0.2^aA^	1.39 ± 0.4^aA^	1.39 ± 0.2^aA^	1.52 ± 0.3^aA^	1.62 ± 0.3^aA^	1.32 ± 0.1^aA^
	72	1.28 ± 0.3^aB^	1.13 ± 0.2^aB^	1.38 ± 0.3^aA^	1.37 ± 0.3^aA^	1.28 ± 0.1^aA^	1.47 ± 0.3^aA^	1.42 ± 0.4^aA^	1.16 ± 0.3^aB^
BCF (Hz)	0	31.2 ± 0.3^aA^	30.2 ± 1.0^aA^	29.9 ± 0.9^aA^	30.1 ± 0.9^aA^	30.2 ± 0.8^aA^	29.9 ± 0.8^aA^	30.0 ± 0.5^aA^	28.2 ± 1.1^aA^
	24	26.7 ± 4.3^aBC^	27.2 ± 1.1^aA^	26.7 ± 1.2^aA^	27.6 ± 1.2^aAB^	28.5 ± 1.3^aA^	28.8 ± 0.9^aA^	29.8 ± 0.9^aA^	29.9 ± 2.6^aAB^
	48	22.5 ± 4.2^abC^	16.5 ± 9.2^bB^	19.1 ± 5.1^bB^	22.3 ± 5.1^bB^	25.7 ± 1.1^aA^	27.5 ± 1.3^aA^	27.7 ± 0.7^aA^	28.2 ± 5.8^aAB^
	72	19.8 ± 4.8^bcC^	4.7 ± 7.8^dC^	14.5 ± 5.8^bB^	19.4 ± 5.8^bcC^	25.1 ± 3.5^abA^	27.1 ± 0.7^aA^	25.5 ± 5.0^abA^	20.9 ± 1.5^abB^

Data (mean ± SD) show the percentage of motile sperm (MOT), percentage of progressively motile sperm (PRG), curvilinear velocity (VCL), straight-linear velocity (VSL), amplitude of lateral head displacement (ALH) and sperm beat cross-frequency (BCF). Different superscript lowercase letters (a, b, c) indicate statistically significant differences between buffers at the same time point; different superscript uppercase letters (A, B, C) indicate statistically significant differences between time points for each buffer (*P* < 0.05)

### Effect of ASP pH

Immediately after dilution (0 h), sperm motility (MOT) was at a high level (range 84.6–94.8%) regardless of the pH (7.0, 7.5, 8.0, 8.5 or 9.0) of the ASP used for preservation (Table 2). After 72-h preservation, the highest MOT value was noted in the ASP at pH 7.5 (74.3%), although a significant difference was found only in comparison to fresh sperm (51.4%; *P* < 0.05). Although progressive motility was greatest (90.7%) in fresh sperm at 0 h, this value differed significantly only from those recorded for ASP at pH 7.0 and 7.5 (Table 2; *P* < 0.05). After 72 h, there were no significant differences in PRG values, regardless of the pH of the ASP used for sperm preservation. Velocity of sperm (VCL and VSL) was at a constantly high level after 24-h preservation in all ASP extenders (Table 2). However, VCL and VSL values decreased significantly in fresh sperm after 48–72 h in comparison to those recorded after 24 and 0 h, and in comparison to the ASP variants. Immediately after dilution (0 h), the lowest ALH values were noted in ASP at pH 7.0 (1.67 μm); this value differed significantly in comparison to that of the other ASP variants (2.49, 2.01, 2.31 and 2.10 μm for pH 7.5, 8.0, 8.5 and 9.0) (Table 2; *P* < 0.05). After 72-h preservation, ALH values were at a constant level in all ASP extenders, although the greatest decrease was observed in ASP at pH 8.0–8.5. The largest drop in BCF values during sperm preservation was found in fresh sperm after 72 h (Table 2). In the tested ASP extenders, BCF values were at a constant level during the first 48-h preservation, with no differences observed among the various pH treatments.

**Table 2:** Motility parameters of common carp *Cyprinus carpio* L. (*n* = 5) sperm during 72-h short-term storage in artificial seminal plasma (ASP: 2 mM CaCl_2_, 1 mM Mg_2_SO_4_, 20 mM Tris, 110 mM NaCl, 40 mM KCl) of varying pH, and for the control group (fresh sperm)

CASA parameters	Time (hours)	Control	pH of ASP				
			7.0	7.5	8.0	8.5	9.0
MOT (%)	0	97.8 ± 1.2^aA^	84.6 ± 9.2^aA^	88.2 ± 6.2^aA^	91.3 ± 6.1^aA^	92.0 ± 6.2^aA^	94.8 ± 3.2^aA^
	24	89.5 ± 5.5^aA^	78.6 ± 6.9^aAB^	82.7 ± 8.2^aA^	89.8 ± 4.1^aA^	83.5 ± 7.7^aA^	87.6 ± 7.2^aAB^
	48	85.6 ± 8.2^aA^	77.6 ± 22.3^aAB^	81.5 ± 17.3^aA^	83.9 ± 9.5^aA^	81.9 ± 5.5^aA^	77.4 ± 13.6^aB^
	72	51.4 ± 18.3^bB^	65.6 ± 6.8^abB^	74.3 ± 2.2^aA^	62.2 ± 5.6^abB^	66.9 ± 10.2^abB^	65.5 ± 14.5^abC^
PRG (%)	0	90.7 ± 4.2^aA^	70.5 ± 15.1^cA^	71.0 ± 14.3^cbA^	86.3 ± 8.5^abcA^	83.7 ± 13.0^abcA^	87.3 ± 5.5^abA^
	24	74.5 ± 14.8^aB^	66.9 ± 24.4^aA^	69.7 ± 9.6^aA^	75.6 ± 7.9^aAB^	69.6 ± 10.3^aAB^	74.6 ± 12.5^aAB^
	48	55.6 ± 20.3^aC^	62.2 ± 12.7^aAB^	66.6 ± 20.5^aA^	65.8 ± 15.3^aB^	63.9 ± 12.6^aBC^	61.3 ± 16.1^aBC^
	72	19.5 ± 11.8^aD^	47.7 ± 10.4^aB^	58.1 ± 4.9^aA^	46.6 ± 6.3^aC^	49.3 ± 7.1^aC^	48.3 ± 11.3^aC^
VCL (μm s^−1^)	0	123.5 ± 13.5^bA^	136.6 ± 6.4^abA^	143.5 ± 7.3^aA^	143.8 ± 8.2^aA^	147.3 ± 13.9^aA^	146.5 ± 12.6^aA^
	24	117.9 ± 13.8^aA^	135.3 ± 8.6^aA^	131.9 ± 9.2^aAB^	132.5 ± 11.4^aAB^	132.5 ± 13.8^aAB^	133.3 ± 6.3^aAB^
	48	88.4 ± 24.6^bB^	131.8 ± 11.4^aA^	129.5 ± 9.4^aAB^	123.6 ± 9.9^aB^	129.1 ± 9.9^aB^	131.1 ± 16.8^aAB^
	72	76.3 ± 28.4^bB^	129.4 ± 14.6^aA^	126.6 ± 16.2^aB^	120.4 ± 12.3^aB^	128.3 ± 15.5^aB^	123.7 ± 21.9^aB^
VSL (μm s^−1^)	0	94.3 ± 7.9^bA^	113.1 ± 4.7^aA^	113.1 ± 4.8^aA^	123.2 ± 3.6^aA^	119.4 ± 13.8^aA^	121.5 ± 3.3^aA^
	24	87.5 ± 10.1^bA^	112.7 ± 10.6^aA^	110.8 ± 14.6^aA^	120.4 ± 12.9^aA^	118.1 ± 4.8^aA^	120.8 ± 11.4^aA^
	48	65.5 ± 18.4^bB^	110.3 ± 13.1^aA^	106.6 ± 7.1^aA^	102.2 ± 7.7^aB^	112.9 ± 14.3^aA^	115.5 ± 17.3^aAB^
	72	63.9 ± 25.7^bB^	108.9 ± 8.2^aA^	103.7 ± 10.3^aA^	101.4 ± 18.2^aB^	111.8 ± 7.1^aA^	106.1 ± 24.4^aB^
ALH (μm)	0	2.28 ± 0.6^abA^	1.67 ± 0.6^bA^	2.49 ± 0.2^aA^	2.01 ± 0.2^abA^	2.31 ± 0.7^abA^	2.10 ± 0.4^abA^
	24	2.05 ± 0.5^aAB^	1.58 ± 0.4^aA^	1.81 ± 0.3^aB^	1.71 ± 0.4^aA^	1.43 ± 0.2^aB^	1.56 ± 0.1^aAB^
	48	1.91 ± 0.3^aAB^	1.55 ± 0.6^aA^	1.46 ± 0.3^aB^	1.64 ± 0.2^aAB^	1.37 ± 0.2^aB^	1.48 ± 0.2^aB^
	72	1.47 ± 0.6^aB^	1.10 ± 0.2^aA^	1.31 ± 0.4^aB^	1.03 ± 0.2^aB^	1.03 ± 0.2^aB^	1.27 ± 0.2^aB^
BCF (Hz)	0	29.5 ± 1.0^aA^	27.8 ± 1.7^aA^	30.3 ± 1.3^aA^	30.4 ± 0.8^aA^	30.6 ± 1.7^aA^	30.6 ± 1.1^aA^
	24	28.7 ± 0.7^aAB^	27.3 ± 2.3^aA^	29.2 ± 0.9^aAB^	29.1 ± 0.7^aA^	28.1 ± 0.9^aAB^	28.5 ± 1.2^aA^
	48	25.3 ± 3.6^aB^	26.7 ± 3.1^aA^	26.7 ± 0.9^aAB^	28.0 ± 3.3^aA^	27.7 ± 0.9^aAB^	26.9 ± 0.9^aA^
	72	18.6 ± 7.3^bC^	26.3 ± 2.6^aA^	26.1 ± 2.1^aB^	26.7 ± 1.5^aA^	25.5 ± 1.0^aB^	26.7 ± 2.2^aA^

Data (mean ± SD) show the percentage of motile sperm (MOT), percentage of progressively motile sperm (PRG), curvilinear velocity (VCL), straight-linear velocity (VSL), amplitude of lateral head displacement (ALH) and sperm beat cross-frequency (BCF). Different superscript lowercase letters (a, b, c) indicate statistically significant differences between buffers at the same time point; different superscript uppercase letters (A, B, C) indicate statistically significant differences between time points for each buffer (*P* < 0.05)

## Discussion

The presented results indicate that the addition of 110 mM NaCl and 40 mM KCl to ASP (2 mM CaCl_2_, 1 mM Mg_2_SO_4_, 20 mM Tris) had a beneficial effect on common carp sperm motility during 72-h short-term storage at 4 °C in × 10 dilution ratio. This effect was observed in ASP with a pH range of 7.0–9.0. Although the highest value of sperm motility was observed at pH 7.5, this difference was not statistically significant. After 72-h preservation, fresh sperm (the control treatment) exhibited lower motility in comparison to that stored in ASP containing various concentrations of NaCl/KCl and with different pH values.

According to Morisawa et al. (1983), the SP of common carp contains not only sodium (75 mM), potassium (82.4 mM) and chlorine (112 mM) ions, but also 2.0 mM Ca^2+^ and 0.8 mM Mg^2+,^ with osmotic pressure around 300 mOsm kg^−1^. In the present study, we therefore used ASP components in similar proportions to their natural concentrations in common carp seminal plasma. In ASP samples supplemented with high concentrations of potassium (150, 130 and 110 mM KCl), sperm motility greatly decreased after 48–72-h preservation. However, this relationship was not observed for ASP supplemented with the same concentration of sodium (150, 130 and 110 mM NaCl), which indicates that K^+^ levels above 100 mM may lead to the disturbance of sperm motility (MOT, PRG, VCL, VSL and ALH) for common carp. The greatest differences in CASA parameter values after 72-h sperm preservation followed the order PRG > VSL > VCL > MOT > BCF; no differences in ALH values were recorded between treatments. Therefore, we conclude that ALH is likely the least sensitive parameter with which to compare sperm quality in the preserved samples of common carp.

In salmonids, the concentration of potassium ions (in combination with osmotic pressure) is the main factor controlling sperm motility. In contrast, common carp sperm motility is inhibited mainly by osmolality (Alavi and Cosson 2006; Redondo-Müller et al. 1991), with sperm exposed to 50 mM KCl found to regain motility potential after a few hours’ incubation (Redondo-Müller et al. 1991). Our results, together with available data in the literature, indicate that a potassium concentration of around 40–50 mM in ASP is essential for maintaining common carp sperm quality during in vitro storage.

In the present study, for ASP samples supplemented with 40/110 or 20/130 mM KCl/NaCl, sperm motility was at a high level after 72-h preservation (79.0 and 72.6%, respectively). In contrast, for ASP supplemented with 40/110 or 20/130 mM NaCl/KCl, sperm motility was significantly lower (31.9 and 21.2%, respectively) after 72-h preservation. A similar tendency was found in all the other tested CASA parameters with the exception of ALH and BCF. In common carp, whereas an inhibitory effect of low KCl concentrations (i.e. 0.5 mM) on sperm motility has not been observed (Linhart et al. 2003), potassium ion concentrations of 150 mM did have such an effect (Perchec et al. 1995). Ravinder et al. (1997) compared several buffers for the short-term storage of common carp sperm, and found that after 24-h preservation, sperm motility was high (88%) when stored in an extender containing 95 mM NaCl, 48 mM KCl, 1.7 mM CaCl_2_ and 25 mM NaHCO_3_ (at pH 8.8 and osmolality 326 mOsm kg^−1^; Yanagimachi 1978). Prolonged sperm storage (up to 84 h), however, resulted in poor motility in this buffer. In our study, the addition of potassium and sodium to ASP at concentrations of 40 and 110 mM, respectively, was the most promising option regarding the short-term storage of common carp sperm. After 72-h preservation, sperm motility in this ASP was around 80%, higher than that recorded in all other treatments. Moreover, our results suggest that the short-term preservation of common carp sperm is possible in appropriate buffers for at least 72 h without any loss in quality. Further studies are required to determine whether the fertility of the preserved sperm also remains unaffected.

The presented data further suggest that common carp sperm can be preserved for 72 h in ASP containing 110 mM NaCl and 40 mM KCl with a relatively wide pH range from 7.0 to 9.0. Previous studies have found that several sperm motility parameters may be influenced by the pH of the immobilising buffer during short-term storage (Perchec et al. 1995). Moreover, a drop in pH may also result in low sperm motility and consequently low fertilisation and hatching rates (Nynca et al. 2012). In goldfish *Carassius auratus* (L.), the preservation of sperm (125 mM NaCl, 0.1 mM CaCl_2_, 20 mM Tris; Saad et al. 1988) for 72 h at pH 6.5 resulted in a significant decrease in sperm viability and motility (25 and 20%, respectively) in comparison to that stored at pH 8.5 (75 and 70%; Chantzaropoulos et al. 2015). Similarly, in common carp, the motility of sperm stored for 24 h at pH 7.8 was greater than that stored at pH 6.0 (Saad et al. 1988). The above results indicate that sperm preservation in an acidic environment may result in damage to motility, potentially caused by a reduction in the intracellular pH of the sperm during storage at a pH below 7.0 (Chantzaropoulos et al. 2015). According to our results, it seems that a preservation buffer pH range of 7.0 to 9.0 is well tolerated by the sperm of the common carp.

In summary, the optimisation of short-term storage techniques and ASP composition is required in order to establish the most promising option for common carp sperm preservation. Such knowledge is also important regarding the physiology of reproduction, i.e. the maturation, storage and ageing of sperm. The results of the present study indicate that for the short-term (72 h) storage of common carp sperm in ASP, the most important factor is the K^+^ concentration, which should be around 40 mM. Further studies are required to identify the osmolality range in which short-term preservation is possible, as well as the pH limits for this procedure and the fertilisation capacity of the stored sperm.
